# Design of Epitopes from Treponema pallidum Lipoprotein Antigens for Syphilis Diagnosis and Treatment Prognosis

**DOI:** 10.1021/acsinfecdis.5c00155

**Published:** 2025-05-23

**Authors:** Letícia Alves Borghezan, Lara Cândida de Sousa Machado, Iara Barreto Neves Oliveira, Mírian Ívens Fagundes, Nicoly Silveira Apolidório, Renato Canevari Dutra da Silva, Victor Garcia Freire, Antonio Augusto Schafer, Rahisa Scussel, Ricardo Andrez Machado-de-Ávila

**Affiliations:** † Programa de Pós-Graduação em Ciências da Saúde, 97853 Universidade do Extremo Sul Catarinense, Universitário, 88806-000 Criciúma, Santa Catarina, Brazil; ‡ Faculdade de Medicina, Universidade de Rio Verde, Fazenda Fontes do Saber, Campus Universitário, 75901-970 Rio Verde, Goiás, Brazil; § Faculdade de Medicina, Universidade de Rio Verde, Avenida T-13 Qd. S-06, Lts.08/13. Setor Bela Vista, 74823-440 Goiânia, Goiás, Brazil; ∥ Programa de Pós-Graduação em Saúde Coletiva, Universidade do Extremo Sul Catarinense, Universitário, 88806-000 Criciúma, Santa Catarina, Brazil

**Keywords:** epitope, treponemal lipoproteins, synthetic peptides

## Abstract

Syphilis, a multistage sexually transmitted infection, causes severe complications if untreated. Accurate diagnosis remains difficult due to the low protein content of Treponema pallidum and in vitro culture difficulty. Advances in immunoproteomics have identified key antigens that enhance diagnostic accuracy. Epitopes of immunodominant antigen proteins Tp0171, Tp0435, Tp0574, Tp0684, and Tp0453 were designed by bioinformatics tools, and mimetic peptides were chemically synthesized. A diagnostic accuracy cross-sectional study was performed to validate a prototype technology as a serodiagnosis platform for syphilis. Five peptides were used as antigens in a peptide-based ELISA against serum samples from syphilis-positive or noninfected patients (*n* = 122). CE*Tp*0435 achieved 100% sensitivity and specificity with a high accuracy. The peptides CE*Tp*0171 and CE*Tp*0574 demonstrated high diagnostic performance, with sensitivity above 83% and specificity >90%. Peptides CE*Tp*0684 and CE*Tp*0453 exhibited sensitivity above 80% and 90%, respectively; CE*Tp*0453 showed reduced specificity (∼66%). The peptides CE*Tp*0435 and CE*Tp*0171 maintained high diagnostic accuracy across syphilis stages, with a sensitivity above 83% and a specificity exceeding 97%. Peptides CE*Tp*0435 and CE*Tp*0171 effectively monitored syphilis treatment, as evidenced by the significant post-treatment decline in serum antibody levels, supporting their potential for evaluating the therapeutic efficacy. We obtained two epitopes mimetic peptides as advantageous antigens for serodiagnosis and treatment monitoring.

## Graphical abstract

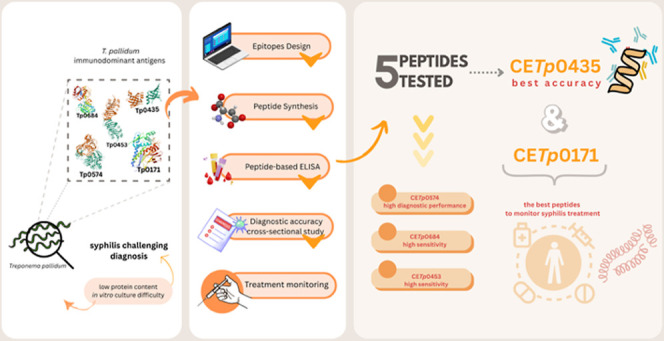

1

Treponema pallidum bacteria are the etiological agent of syphilis, a multistage chronic sexually transmitted infection (STI), which leads to significant complications when untreated. Syphilis has been shown to facilitate the acquisition and transmission of the human immunodeficiency virus (HIV) and other STIs, such as gonorrhea and chlamydia.^1^ Syphilis progresses through four stages—primary, secondary, latent, and tertiary—each with distinct clinical features. Primary syphilis is marked by a painless ulcer (chancre), which may be unnoticed in hidden areas such as the rectum. Secondary syphilis presents as a nonitchy rash, often on the palms and soles, and may include systemic symptoms such as fever, lymphadenopathy, and general malaise. Latent syphilis lacks symptoms but can progress to tertiary syphilis, which may cause severe neurological or cardiovascular complications.^2^

The World Health Organization (WHO), in 2022, worldwide estimated that approximately 8 million individuals aged 15–49 years old contracted syphilis. Several countries reported an increase in cases of syphilis after the COVID-19 pandemic.^1^ In 2022, a total of 35,391 confirmed cases of syphilis were reported across 29 European Union/European Economic Area (EU/EEA) Member States. This reflected a 34% increase in the crude notification rate compared to 2021.^3^ From 2020 to 2022, incident cases of syphilis in the Americas increased by 30%.^2^ Between 2010 and 2024, Brazil reported 1,538,525 cases of acquired syphilis, 713,167 cases of syphilis in pregnant women, 344,978 cases of congenital syphilis, and 3,554 deaths from congenital syphilis in infants under one year of age.^4^ Clinical evolution and untreated syphilis risk underscoring the need for effective individual detection strategies. Yet access to essential health supplies at primary healthcare levels remains insufficient in many low- and middle-income countries. Inadequate surveillance and limited healthcare access in low- and middle-income countries likely result in underreported syphilis cases, delaying progress toward the targets of the WHO,^5^ which are aligned with the Sustainable Development Goals of the United Nations’ 2030 Agenda. The global rise in syphilis cases, particularly in the WHO African Region and the Region of the Americas, underscores the urgent need for improved diagnostic tools.^1^

Despite advances over the last decades in the prevention and treatment of syphilis, accurate diagnosis remains urgent and challenging. This difficulty arises primarily from the experimental limitations associated with T. pallidum, the etiological agent of syphilis. Although some studies described in the literature have already managed to cultivate a bacterium in vitro on a cell culture-based system and generate results from these studies, this methodology has some limitations.^6,7^ The yield of treponemal cells from in vitro culture remains limited compared to other bacteria with significantly shorter generation times that can be propagated in axenic cultures.^8^ Furthermore, they depend exclusively on a mammalian host for sustained growth and viability.^9–11^ Consequently, T. pallidum must be propagated through experimental infections in rabbits.^9,11^ Another obstacle faced in T. pallidum research, mainly for the identification of antigens, is the bacterium’s fragile outer membrane and low protein content.^9,12^ Thus, comprehensive proteome-wide analysis of the syphilis spirochete presents technical challenges due to the sample complexity, limited bacterial availability, and the fragility of its cell envelope.^13^ Advances in genomic and recent proteomic approaches to T. pallidum research using experimental rabbit models are the leading basis for immunodominant antigens identification.^9–12,14–17^

Currently, syphilis diagnosis is performed by a costly and complex process that depends on the combination of several laboratory tests, the patient’s clinical and sexual history, and clinical semiology and anamnesis. The syphilis laboratory test predominantly relies on serological testing, which are nontreponemal tests (NTTs) and treponemal tests (TTs).^1,18,19^ These tests should be used in combination, following the traditional syphilis screening algorithm (initial screening with NTTs) or the reverse syphilis screening algorithm (initial screening with TTs), since leaning on a single reactive serologic test result can misclassify a patient’s syphilis status.^20,21^ NTTs are mainly lipoidal serologic tests, such as the venereal disease research laboratory (VDRL), which identify antibodies targeting lipoidal molecules released by both damaged host cells and T. pallidum during active infection. The relatively variable sensitivity of these assays (ranging from 74% to 99%) can occasionally result in misdiagnoses in patients with primary syphilis.^15^

Due to some drawbacks of NTTs, such as unusual increases post-treatment or decreases in antibodies titers observed in untreated individuals,^18^ several TTs diagnostic platforms have been developed. Recombinant T. pallidum lipoproteins, identified as highly specific for T. pallidum subspecies, immunodominant, and among the protein content of T. pallidum already identified, are the most expressed and have been tested as diagnosis antigens. Previous immunoproteomic studies^22,23^ pointed out some inner membrane lipoproteins, mainly Tp0171 (TpN15), Tp0435 (TpN17), and Tp0574 (TpN47), as reactive with sera from patients and infected rabbits at all stages of syphilis, even the early stages. Hence, these antigens are currently the most commonly used in the serodiagnosis of syphilis, as they elicit highly specific immune responses that are applied in treponemal tests.^12,15,24,25^ Also, the TpN15-TpN17-TpN47 fusion protein was previously proven to be a highly sensitive and specific immune detection tool for T. pallidum.^26^ Another antigen of particular interest, TP0453 membrane protein, was reactive with sera from patients with primary syphilis, suggesting that antigen might be valuable in early diagnostic studies.^22^ However, there is no consensus on which antigens have the best serodiagnostic performance for syphilis diagnosis and treatment prognosis.^27^ An accurate and simple approach to the diagnosis of syphilis and then a tool for assessing the response to treatment are lacking.

In this sense, bioinformatics tools have been rising in the development of new diagnostic strategies. The identification of epitopes by bioinformatics has proven to be a useful tool for developing immunoassay antigens for the accurate detection of specific antibodies.^16,28–30^ Most antigenic determinants recognized by B cells and antibodies are conformational epitopes. These epitopes comprise noncontiguous amino acid residues in the primary protein sequence, which form specific regions upon protein folding, reflecting the native structural conformation of the protein.^31^ Such conformational epitopes are particularly effective as antigen coatings in ELISA as serodiagnosis platforms.^32,33^

Thus, in this study, our main aim was to validate by a diagnostic accuracy cross-sectional study a prototype diagnostic platform peptide-based enzyme-linked immunosorbent assay using five highly immunogenic T. pallidum protein epitopes predicted by bioinformatic tools. We also further evaluate the effectiveness of this platform peptide-based in surveilling syphilis treatment efficacy.

## 2 Results

### 2.1 Participants Epidemiological Profile

Table 1 summarizes the sociodemographic and clinical characteristics of the participants. A total of 51.6% were female. Regarding skin color, 51.2% self-identified as brown, followed by 24.8% as white, 17.4% as yellow, and 6.6% as black. Most participants were HIV-negative (93.4%), and 63.9% tested positive for syphilis. Among those diagnosed with syphilis, 67.5% were in the latent stage, 17.5% were in the primary stage, and 15.0% were in the secondary stage. Additionally, 28.9% were undergoing syphilis treatment at the time of data collection, while 71.1% had completed treatment. Of those participants who completed the treatment, 25.93% had a syphilis history, and remained seropositive, presenting reactivity in TT monitoring tests, i.e., chemiluminescence but without reactivity for VDRL. The mean age of participants was 32.46 years (SD = 11.42); data is not presented in the table.

**Table 1:** Sociodemographic and Clinical Characteristics of the Study Participants^a^

	*n*	%
**gender**		
male	59	48.4
female	63	51.6
**skin color**		
white	30	24.8
black	8	6.6
brown	62	51.2
yellow	21	17.4
**HIV status**		
negative	114	93.4
positive	8	6.6
**syphilis status**		
negative	44	36.1
positive	78	63.9
**syphilis stage**		
primary	7	17.5
secondary	6	15.0
latent	27	67.5
**syphilis treatment**		
ongoing treatment	11	28.9
post-treatment	27	71.1

a Rio Verde, Brazil, 2022/2023. (*n* = 122).

### 2.2 Syphilis Serodiagnosis Platform Index Test Validation

#### 2.2.1 Epitope of Treponemal Antigens Proteins

The epitopes were predicted following two different algorithms from IEDB. Prediction results were integrated and utilized to design conformational and linear epitopes within the three-dimensional structures utilized by Swiss-PDB-Viewer. For each protein, Tp0171 (PDB: 4XDU), Tp0435 (PDB: 4UQ3), Tp0574 (PDB: 1O75), Tp0453 (PDB: 3K8J), and Tp0684 (PDB: 5JX2), an epitope-peptide was predicted as illustrated in Figure 2. Table 2 shows the amino acid sequence obtained from those epitopes from T. pallidum immunogenic proteins (CETp) and the *physicochemical properties* of their synthetic peptides.

**Table 2:** Sequences of Epitopes according to the Amino Acid Position in T. pallidum Proteins and Physicochemical Properties of Their Synthetic Peptides

peptide	peptide sequence in protein position	isotropic mass (g/mol)	isoelectric point (pH)	water solubility
CE*Tp*0171	Lys_225_Gln_224_Gly_223_Thr_222_Gly_221_His_220_Pro_219_Asp_118_Arg_217_Tyr_243_Phe_246_Phe_247_Gln_248_Arg_249_Asp_250_Ala_115_Ala_116_Val_117_Pro_118_Asp_119_Pro_120_Asp_121_Ala_122_Leu_123_Lys_124_Glu_125_	2956.27	9.70	good
CE*Tp*0435	Lys_10_Ala_11_Lys_12_Ala_13_Glu_14_Lys_21_Ala_45_Asp_46_Gly_47_Thr_48_Ala_49_Gln_50_Arg_68_Tyr_67_Thr_66_Leu_65_Pro_64_	1848.09	9.53	good
CE*Tp*0574	Val_97_Leu_98_Ser_99_Lys_100_Gln_101_Glu_102_Thr_103_Glu_104_Asp_105_Ser_106_Arg_107_Gly_108_Arg_109_Lys_110_Lys_111_Trp_112_Glu_113_Tyr_114_Glu_115_Thr_116_Asp_117_Pro_118_Ser_119_Val_120_	2867.12	6.28	good
CE*Tp*0684	Asn_272_Ser_273_Glu_274_Val_275_Thr_276_Ser_277_Ala_278_Asn_279_Trp_280_Lys_281_Glu_282_Tyr_283_Thr_284_Arg_285_Gly_286_Ala_287_	1812.91	6.14	poor
CE*Tp*0453	Val_163_Asn_164_Pro_165_Asp_166_Arg_167_Pro_168_Gln_169_Leu_170_Pro_171_Pro_172_Arg_173_Phe_174_Glu_175_Lys_176_Glu_177_Cys_178_Thr_179_Ser_180_Glu_181_Gly_182_Thr_183_	2400.65	4.87	good

**Figure 1:**
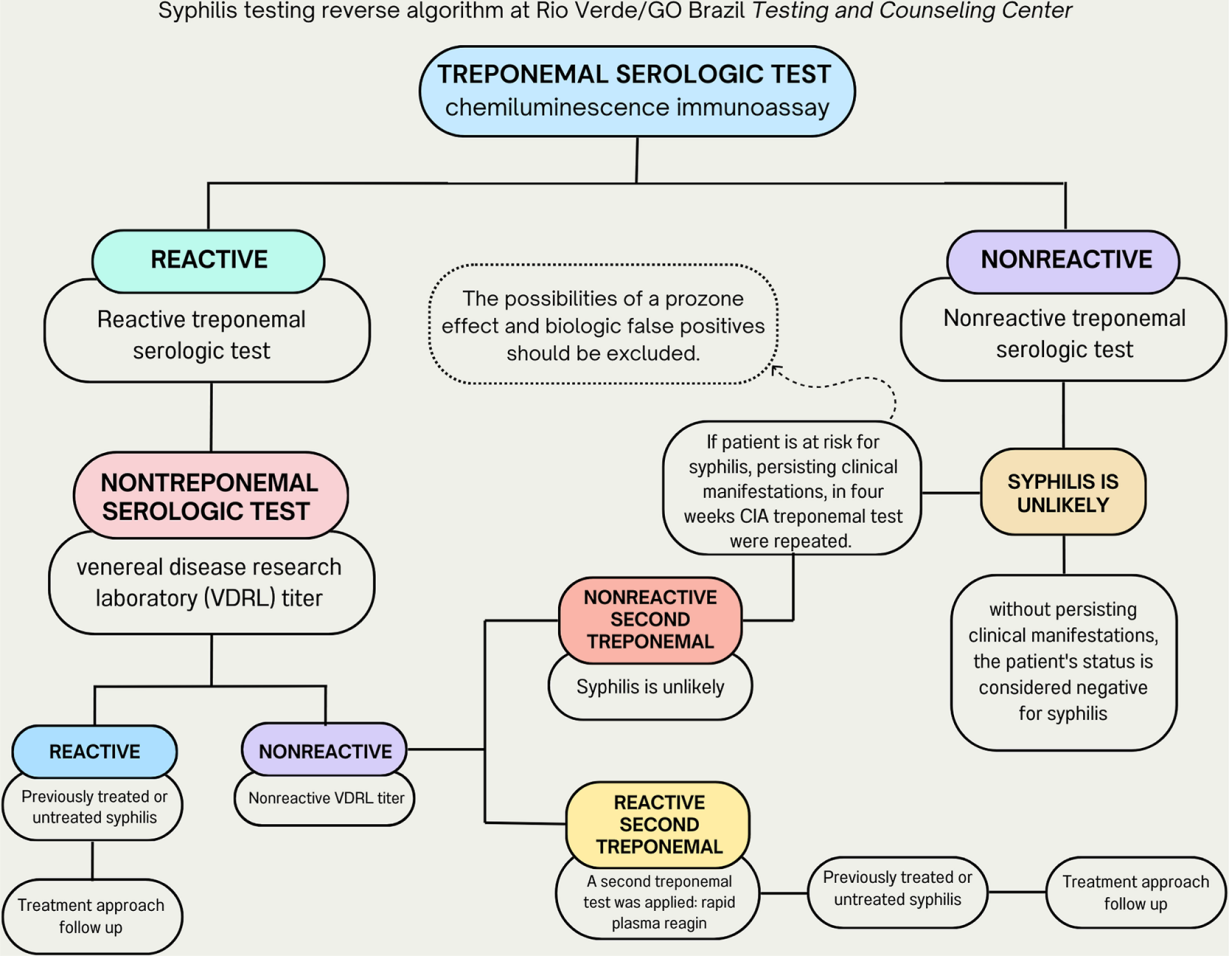
Algorithm adopted for syphilis screening at the Rio Verde/GO—Brazil, CTA, using a reverse approach according to the Technical Manual for Syphilis Diagnosis of the Brazilian Ministry of Health.

**Figure 2:**
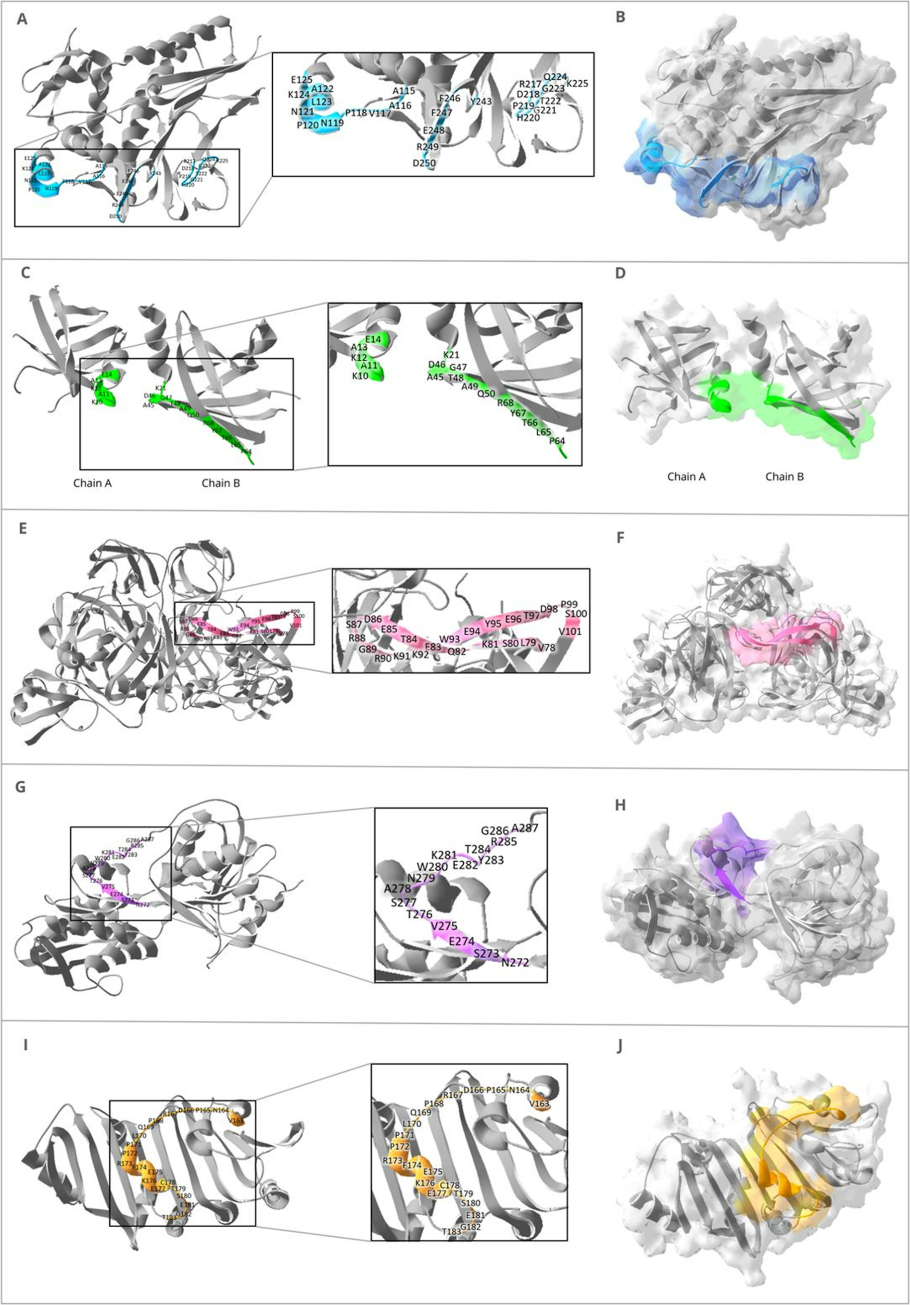
Epitopes on T. pallidum proteins on 3D structures. Peptide CE*Tp*0171 is highlighted in light blue in the ribbon diagram (A) and on the molecular surface (B). Peptide CE*Tp*0435 is highlighted in green in the ribbon diagram (C) and on the molecular surface (D). The peptide CE*Tp*0574 is highlighted in pink in the ribbon diagram (E) and on the molecular surface (F). Peptide CE*Tp*0684 is highlighted in purple in the ribbon diagram (G) and on the molecular surface (H). Finally, peptide CE*Tp*0453 is highlighted in orange in the ribbon diagram (I) and on the molecular surface (J).

Following peptide chemical synthesis, they were characterized by mass spectrometry to ensure their identity by the mass-to-charge ratio (*m*/*z*), confirming their molecular mass by the fragmentation pattern. The precursor ion was fragmented using argon, and confirmation of amino acid sequences was acquired by matching the fragmentation profile spectrum (data not shown). The monoisotopic mass and fragmentation pattern of each peptide were determined using the *Molecular Weight Calculator tool* from MassLynx software, as shown in Table S1.

#### 2.2.2 Treponemal Epitope in Peptide-Based ELISA Diagnosis Validation

Peptide-based ELISA was performed to assess specific antibody detection against treponemal epitopes, using patients’ sera samples (*n* = 40) compared to those from noninfected donors (*n* = 44), considered negative controls. All peptides were analyzed individually for polyvalent IgM, IgA, and IgG detection. The area under the ROC curve (AUC) was used to evaluate the accuracy of the diagnostic performance of the peptide-based ELISA by analyzing its sensitivity and specificity. The ROC curves for all peptide-based ELISAs are presented in Figure 3, *while* Table 3 summarizes the cutoff values and ROC parameters. CETp0171 and CETp0435 were able to distinctly recognize sera samples from syphilis-positive patients, Figure 3A,C. The OD492 values were considered positive when greater than the OD492 mean from negative control plus 2.5 SD. Peptides CETp0171 and CETp0435 demonstrated optimal diagnostic performance, AUC = 0.98 and 1.00, respectively, achieving a specificity of 92.31 and 100%, and a sensitivity of 94.29 and 100%, respectively, effectively differentiating between positive and negative samples (Figure 3A–D, Table 3). *These results showed that these peptides* exhibited the best performance among all the ones evaluated. The peptide CETp0574 exhibited a sensitivity of 83.33% and specificity exceeding 90% (Figure 3F and Table 3), which could be considered a satisfactory diagnosis tool. Peptide CETp0684 showed AUC values ≥ 0.84, with sensitivity ≥ 80% and specificity ≥ 70% (Figure 3H, Table 3). Peptide CETp0453 exhibited a sensitivity exceeding 90% and showed AUC values ≥ 0.84; however, its specificity was approximately 66% (Figure 3J and Table 3), limiting its ability to accurately detect positive samples without potential cross-reactivity. Despite these favorable measurements, the peptides CETp0574 (Figure 3E), CETp0684 (Figure 3G), and CETp0453 (Figure 3I) were unable to completely differentiate positive from negative samples.

**Table 3:** ROC Curve Parameters for Polyvalent Specific Detection (IgA, IgM, and IgG) Using Peptide-Based ELISA as a Serodiagnosis Tool for Syphilis^a^

antigen	parameters									
	AUC	95% CI	cutoff	Se (%)	95% IC	Sp (%)	95% CI	LR+	PPV	NPV
CE*Tp*0171	0.98	0.96 a 1.00	<0.454	94.29	81.39–98.98	92.31	79.68–97.35	12.26	1.0	0.87
CE*Tp*0435	1.00	1.00 a 1.00	<0.402	100.0	91.62–100.0	100.0	91.62–100.0	42.00	1.00	1.00
CE*Tp*0574	0.93	0.87 a 0.99	<1.429	83.33	69.40–91.68	93.10	78.04–98.77	12.08	0.89	0.55
CE*Tp*0684	0.84	0.75 a 0.93	<1.169	83.72	70.03–91.88	74.36	58.92–79.37	2.512	0.91	0.65
CE*Tp*0453	0.84	0.76 a 0.92	<0.827	90.48	77.93–96.23	66.67	51.55–78.99	2.714	0.98	0.62

a Sera samples from patients with syphilis (*n* = 40) and uninfected individuals (*n* = 44) were used in peptide-based ELISA. The statistical analysis of the ROC (receiver operating characteristic) curve, obtaining a value of *p* < 0.0001, to determine the accuracy through the area under the ROC curve (AUC), sensitivity (Se), and specificity (Sp), with confidence interval (CI 95%). The positive likelihood ratio (LR+) was obtained through ROC analysis by the sensitivity ratio by 1-specificity. The positive predictive values (PPV) and negative predictive values (NPV) were obtained by the mathematical correlation between true positives and false positives and by true negatives and false negatives, respectively; using the standard 2 × 2 contingency table method.

**Figure 3:**
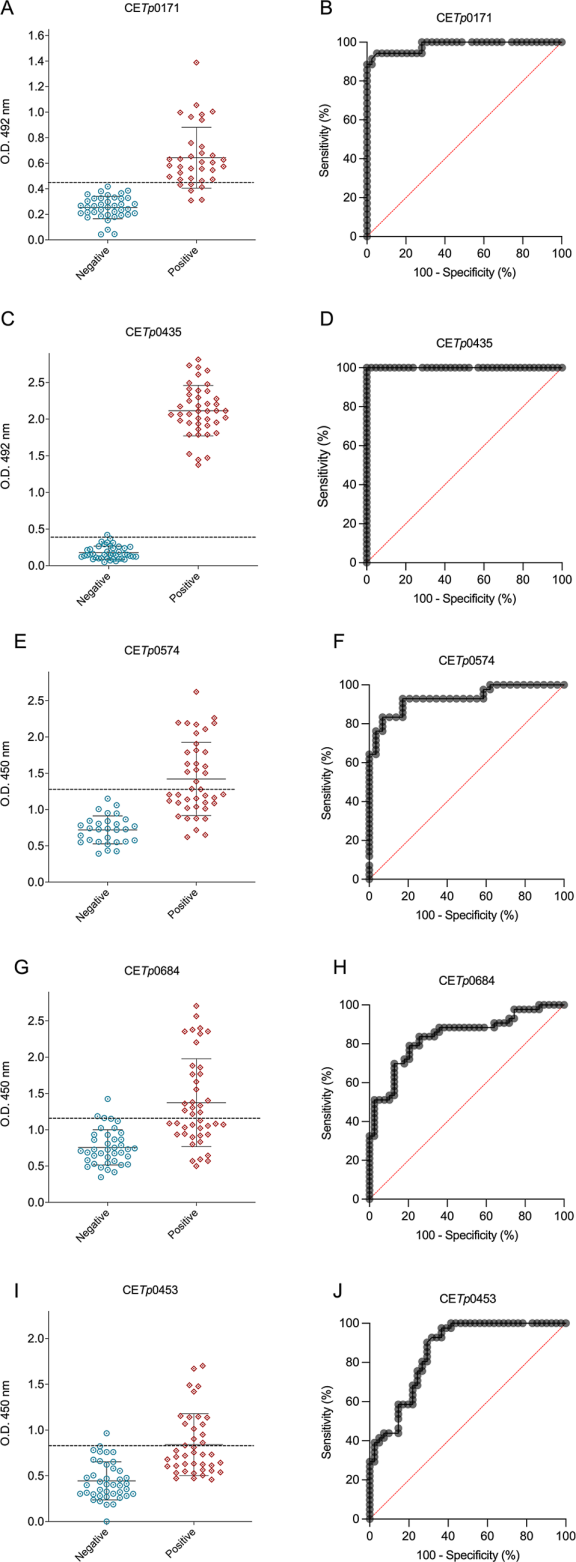
Serological reactivity using synthetic peptides as antigens for serodiagnosis of syphilis and its ROC curve. Antigen (peptide) CE*Tp*0171 serological reactivity (A) and its ROC curve (B); antigen (peptide) CE*Tp*0435 serological reactivity (C) and its ROC curve (D); antigen (peptide) CE*Tp*0574 serological reactivity (E) and its ROC curve (F); antigen (peptide) CE*Tp*0684 serological reactivity (G) and its ROC curve (H); and CE*Tp*0453 serological reactivity (I) and its ROC curve (J). The polyvalent conjugated was diluted 1:5000 in 0.5% BSA in PBS-T. Light blue dots represent the optical density values for each negative sample, and red ones represent the optical density values for each positive sample. These values were used to determine receiver operator curves (ROC). The cutoff values (dotted lines in black) for positive (syphilis serum samples (*n* = 40)) and negative (noninfected donors (*n* = 44)) samples were determined by the mean of negative control samples (noninfected) plus 2.5 SD.

Based on validation by ROC analysis of those five antigens (peptides) and considering syphilis is a multistage chronic sexually transmitted infection (STI), which progresses through stages such as primary, secondary, and latent, the samples were also evaluated by clinical stages of each participant at the sample collection moment. In this sense, CE*Tp*0171 shows a satisfactory tool to detect secondary conditions and a good one to detect primary and latent conditions (Figure 4A). The ROC curve only for secondary samples exhibits AUC = 1.0 and a sensibility and specificity of 100% (Figure 4C). This peptide also exhibits AUC = 0.9856, a sensibility of 87.50%, and a specificity of 97.44% for the primary stage (Figure 4B), and AUC = 0,9808, a sensibility of 83.33%, and a specificity of 97.44% for the latent stage (Figure 4D).

**Figure 4:**
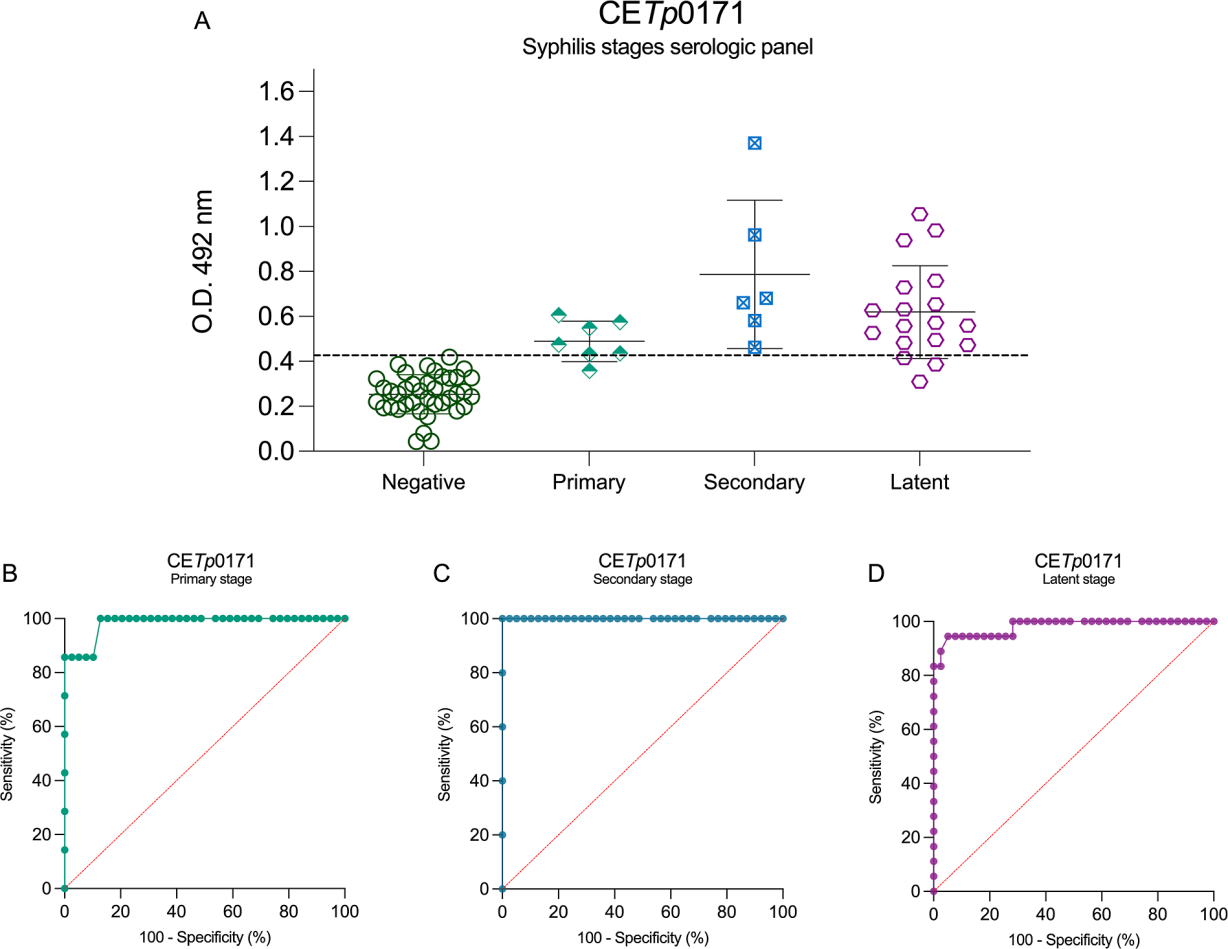
Syphilis stage serological panel using CE*Tp*0171 peptide as antigen and its ROC curves. CE*Tp*0171 serological panel of syphilis multistage (A), primary-stage ROC curve (B), secondary-stage ROC curve (C), and latent-stage ROC curve (D). The cutoff values (dotted lines in black) for positive (syphilis serum samples (*n* = 40)) and negative (noninfected donors (*n* = 44)) samples were determined by the mean of negative control samples (noninfected) plus 2.5 SD.

CE*Tp*0435 demonstrated superior performance, exhibiting highly accurate ratios on the detection of all stages of syphilis (Figure 5A). The ROC curves for the primary (Figure 5B), secondary (Figure 5C), and latent stages (Figure 5D) exhibit AUC = 1.0 and a sensibility and specificity of 100%.

**Figure 5:**
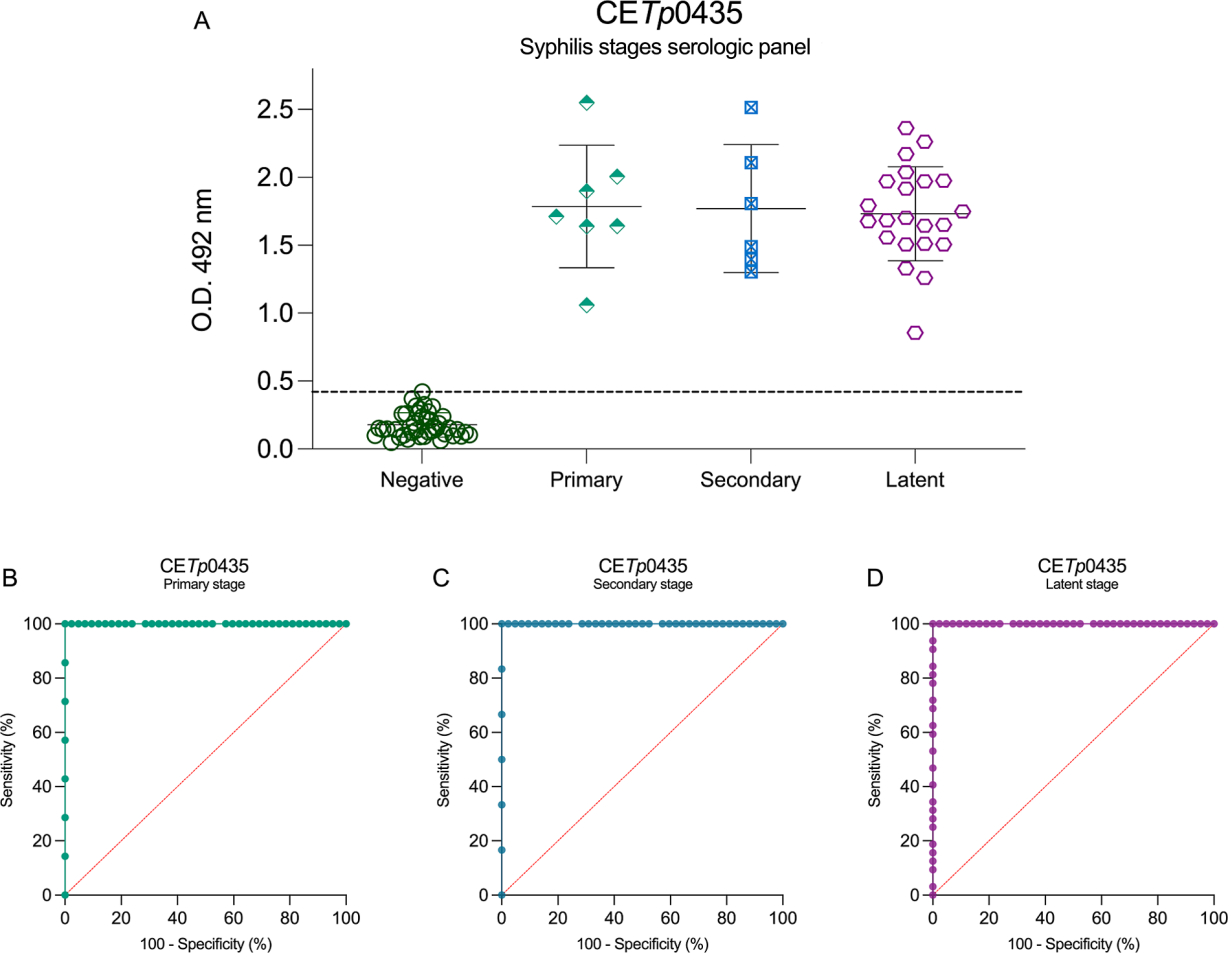
Syphilis stage serological panel using CE*Tp*0435 peptide as antigen and its ROC curves. CE*Tp*0435 serological panel of syphilis multistage (A), primary-stage ROC curve (B), secondary-stage ROC curve (C), and latent-stage ROC curve (D). The cutoff values (dotted lines in black) for positive (syphilis serum samples (*n* = 40)) and negative (noninfected donors (*n* = 44)) samples were determined by the mean of negative control samples (noninfected) plus 2.5 SD.

CE*Tp*0574 demonstrates some errors in accurately detecting positive rates across all stages of syphilis (Figure 6A). Despite this, the ROC curve for primary-stage samples showed an AUC of 0.9754, with 85.71% sensitivity and 100% specificity (Figure 6B). For the secondary stage, this peptide achieved an AUC of 0.8756, with 83.33% sensitivity and 96.55% specificity (Figure 6C). Similarly, for the latent stage, the AUC was 0.8726, with a sensitivity of 65.22% and a specificity of 96.55% (Figure 6D).

**Figure 6:**
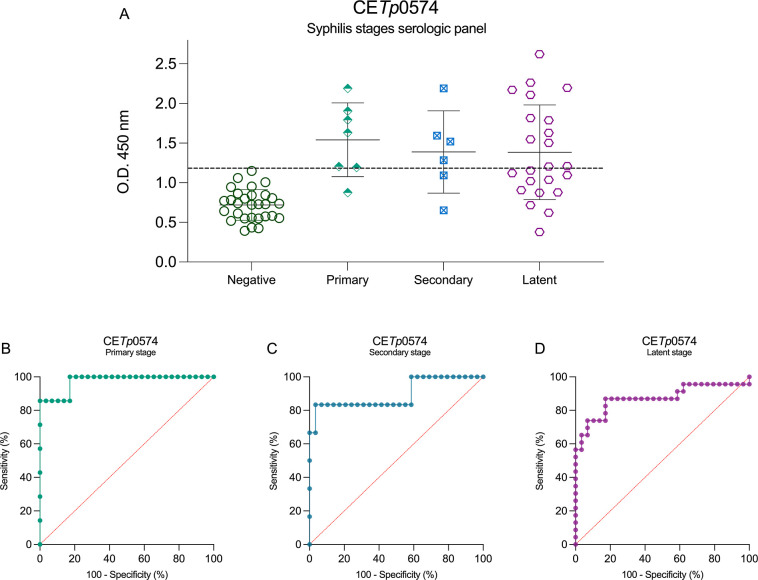
Syphilis stage serological panel using CE*Tp*0574 peptide as antigen and its ROC curves. CE*Tp*0574 serological panel of syphilis multistage (A), primary-stage ROC curve (B), secondary-stage ROC curve (C), and latent-stage ROC curve (D). The cutoff values (dotted lines in black) for positive (syphilis serum samples (*n* = 40)) and negative (noninfected donors (*n* = 44)) samples were determined by the mean of negative control samples (noninfected) plus 2.5 SD.

CE*Tp*0684 also demonstrated a questionable capacity for accurately distinguishing positive samples across all stages of syphilis (Figure 7A). Its ROC curve of the primary stage presented an AUC of 0.9048, with a 71.43% sensitivity and 97.44% specificity (Figure 7B). In the secondary stage, this peptide achieved an AUC of 0.800, with 60.00% sensitivity and 87.18% specificity (Figure 7C). Similarly, for the latent stage, the AUC was 0.8310 but with a sensitivity of 86.36% and a specificity of 79.49% (Figure 7D).

**Figure 7:**
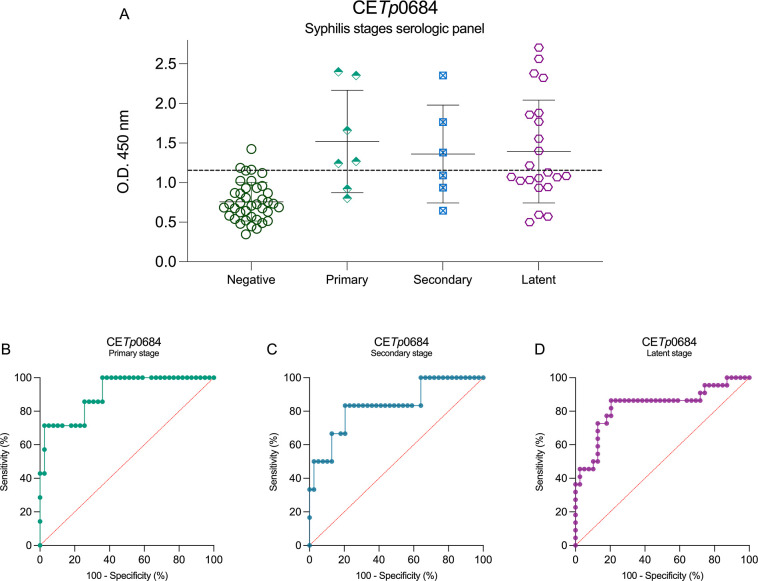
Syphilis stage serological panel using CE*Tp*0684 peptide as antigen and its ROC curves. CE*Tp*0684 serological panel of syphilis multistage (A), primary-stage ROC curve (B), secondary-stage ROC curve (C), and latent-stage ROC curve (D). The cutoff values (dotted lines in black) for positive (syphilis serum samples (*n* = 40)) and negative (noninfected donors (*n* = 44)) samples were determined by the mean of negative control samples (noninfected) plus 2.5 SD.

CE*Tp*0453 demonstrated inferior performance, exhibiting misdiagnoses across all stages of syphilis, and questionable capacity for accurately distinguishing positives from negative samples (Figure 8A). The CE*Tp*0453 ROC curve of the primary stage shows an AUC = 0.8968, with 66.67% sensitivity and 85.71% specificity (Figure 8B). This peptide achieved an AUC of 0.8476, with 60.00% sensitivity and 83.33% specificity for the secondary stage (Figure 8C). The latent stage exhibited the worst performance, with AUC = 0.7759, with a sensitivity of 69.57% and a specificity of 69.05% (Figure 8D).

**Figure 8:**
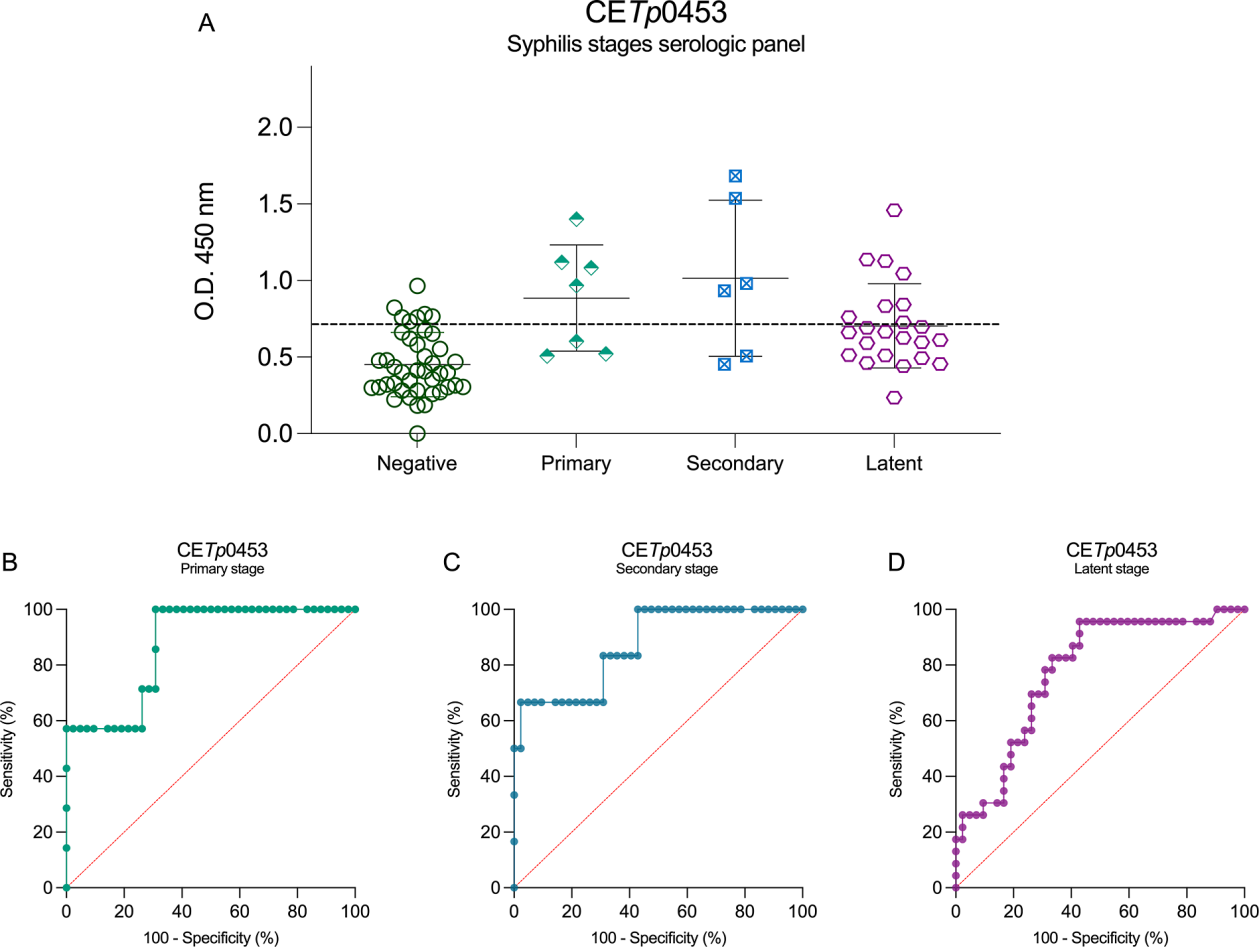
Syphilis stage serological panel using CE*Tp*0453 peptide as antigen and its ROC curves. CE*Tp*0453 serological panel of syphilis multistage (A), primary-stage ROC curve (B), secondary-stage ROC curve (C), and latent-stage ROC curve (D). The cutoff values (dotted lines in black) for positive (syphilis serum samples (*n* = 40)) and negative (noninfected donors (*n* = 44)) samples were determined by the mean of negative control samples (noninfected) plus 2.5 SD.

Since peptides CE*Tp*0171 and CE*Tp*0435 demonstrated optimal diagnostic performance and accurately detected all stages of syphilis, they were used to evaluate the effectiveness of our platform peptide-based system in surveilling syphilis treatment efficacy. A total of 122 unpaired follow-up samples were collected from participants classified as noninfected (*n* = 44); pretreatment (*n* = 40); including 7 with primary-stage syphilis, 6 with secondary-stage syphilis, and 27 with latent syphilis; ongoing treatment (*n* = 11); and post-treatment (*n* = 27; of which *n* = 7 had a history of syphilis). The levels of serum antibodies against CE*Tp*0171 (Figure 9A) and CE*Tp*0435 (Figure 9B), tested by peptide-based ELISA, were significantly higher in pretreatment compared to ongoing and post-treatment (Figure 9, *p* < 0.0001). The pretreatment group presented higher (*p* < 0.0001) levels of serum antibodies against CE*Tp*0171 (Figure 9A) and CE*Tp*0435 (Figure 9B) when compared to negative samples. No statistically significant differences were observed among negative, ongoing-treatment, and post-treatment samples.

**Figure 9:**
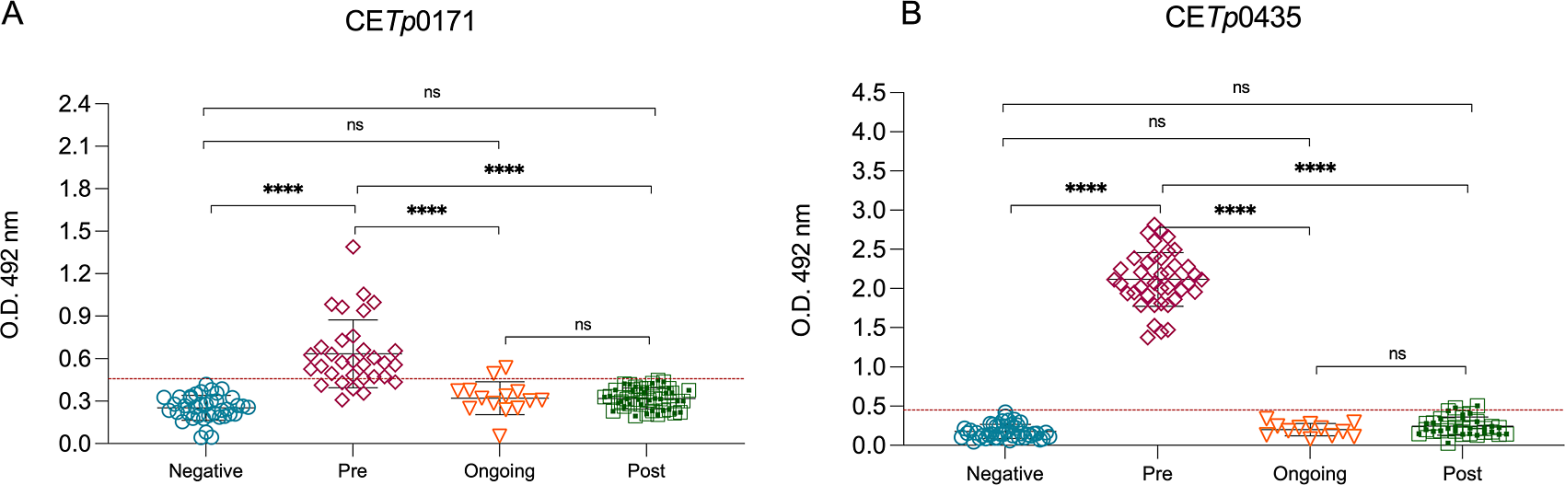
Surveilling syphilis treatment efficacy using antibody levels across treatment phases. Antibody levels against CE*Tp*0171 (A) and CE*Tp*0435 (B) across treatment phases. Data are presented as mean ± standard deviation (SD). Data normality was assessed using the Shapiro–Wilk test. For comparisons between groups, Welch’s ANOVA was performed, due to violation of normality and homogeneity of variances, as indicated by the Brown–Forsythe test (*p* < 0.0001). Multiple comparisons between the control group and the experimental groups were performed using the Dunnett T3 test, adjusting *p* values for multiple comparisons. Statistically significant differences are indicated by asterisks: ****p ≤ 0.0001, ns means nonstatistically significant difference.

## 3 Discussion

Previous studies have reported that several inner membrane lipoproteins of T. pallidum such as TP0171 (Tp15), TP0435 (Tp17), TP0574 (Tp47), TP0684, and TP0453 have strong immunogenicity when evaluated against human sera and infected rabbit sera.^9,10,15^ These findings suggest that these lipoproteins are the main virulence factors and are also recognized as coating antigens in treponemal serodiagnosis tests for syphilis.^34–38^ The proteins Tp0171 (TpN15), Tp0435 (TpN17), Tp0574 (TpN47), Tp0684, and Tp0453 are named based on their respective gene codes in T. pallidum.^14^ Each protein is identified by the *Tp* prefix, indicating that it originates from T. pallidum, followed by a specific gene number. These outer-membrane proteins (OMP) are also named by designations, such as TpN15 or Tp15 for Tp0171, due to its putative gene function, as a 15 kDa lipoprotein; Tp0435 is a 17 kDa lipoprotein known as TpN17 or Tp17; Tp0574 is a 47 kDa lipoprotein designed as TpN47 or Tp47.^15^ These proteins involve various functions related to the bacterium’s survival, virulence, and host interactions.^12^ Tp0684 and Tp0453 are also part of this group, likely playing roles in similar processes. The OMPs are the major targets of host adhesion and the immune response.^39^

Optimization of bacterium sample preparation and mass spectrometry-based proteomics pipeline has enabled the determination of treponemal humoral response profiling, identifying potential antigens capable of enhancing the performance and capabilities of treponemal tests.^11,12,15^ Nevertheless, these approaches are costly and require animals to obtain T. pallidum samples. Therefore, bioinformatic methods have been used to characterize T. pallidum proteins, including lipoprotein characterization.^40^

Based on that, we designed conformational epitopes from Tp0171 and Tp0435 and linear epitopes from Tp0574, Tp0684, and Tp0453 lipoproteins using pipeline bioinformatic tools. We have previously developed peptide-based ELISA that is highly sensitive and accurate in detecting anti-SARS-CoV-2 humoral response using this bioinformatic pipeline to build highly efficient antigens.^32^ Faced on recombinant proteins, peptides offer advantages when employed on serodiagnosis as they combine epitope regions while preserving the protein structural conformation,^32^ potentially enhancing interaction with specific antibodies and reducing cross-reactivity, as reported previously for Lyme disease and syphilis.^41^

The CE*Tp*0435 peptide presented a preeminent performance among all tested antigens. CE*Tp*0435 could differentiate the true positives from negatives, obtaining 100% sensitivity, 100% specificity, and an AUC = 1.0. Although the CE*Tp*0171 and CE*Tp*574 peptides were not able to correctly differ all true positives from negatives samples, CE*Tp*0171 exhibited a sensitivity of 94.29%, specificity of 92.31%, and AUC = 0.98, while CE*Tp*0574 presented 83.33% sensitivity, 93.10% specificity, and AUC = 0.93 (Table 3 and Figure 3A,E). Peptide CE*Tp*0684 demonstrated AUC ≥0.84, sensitivity ≥80%, and specificity ≥70% (Figure 3H, Table 3). CE*Tp*0453 showed high sensitivity (>90%) and AUC ≥0.84 but limited specificity (∼66%), reducing its accuracy due to potential cross-reactivity (Figure 3J, Table 3).

The superior performance of the CE*Tp*0435 peptide, followed by CETp0171 and CETp0574, may be related to their source proteins, which have been previously identified as immunodominant antigens. In immunoproteomic studies, Tp0435, Tp0171, and Tp0574 showed strong reactivity with sera from patients at all stages of syphilis—primary, secondary, and latent^9,10,12,15^ corroborating with our finds. An immune multiplex detection screening for syphilis was based on (IgG) specifically reacting with T. pallidum recombinant proteins Tp0171, Tp0435, and Tp0574, and new synthetic proteins Tp0684 and Tp0453 were carried out on 85 sera from people with previously diagnosed syphilis and 46 noninfected donor sera.^38^ Following our results, the diagnostic efficiency of the immune multiplex was Tp0435 (93,6%) > Tp0171 (81.8%) > Tp0574 (74.8%). Nonetheless, Tp0684 presented 100% specificity and 16.9% sensibility, while the results of our study showed higher sensitivity (83.72%) and worse specificity (74.36%). In general terms, peptide CE*Tp*0684 exhibited the best accuracy performance (84%) when compared with Tp0684 (46.7%). Tp453 presented the best performance from this immune multiplex platform, presenting 100% specificity and 97.5% sensibility,^38^ but the results of our study for peptide CE*Tp*0453 showed limited specificity (∼66%).

This specificity of CETp0453 might be associated with its physicochemical properties. Coating buffers stabilize the antigen (i.e., peptide) and maximize adsorption to the high-binding polystyrene plate.^42^ The carbonate buffer with pH 9.6 is more usual^43–47^ as a coating buffer. Coating is a crucial step for ELISA performance and is directly dependent on the peptide adsorption process. Thus, some studies investigating the driving forces for protein/peptide adsorption onto surfaces such as high-binding polystyrene emphasize the importance of enthalpic contributions, including van der Waals forces, electrical double-layer interactions, and hydrophobic effects,^48^ beyond solution pH, temperature, molecular weight, isoelectric point, and chemical properties of peptides.^49,50^ Based on that, this peptide, which has an isoelectric point of 4.87 and was diluted in carbonate buffer with pH 9.6, could be charged in a way that interferes with the adsorption process since the peptides that obtained the best results in our study have an isoelectric point close to 9.6, see Table 2. Consequently, the CE*Tp*0453 peptide may have its sensitization reduced due to its isoelectric point.

A recent study evaluated the diagnostic performance of three LISA tests screening 261 serum samples, used TP15 (Tp0171), TP17 (Tp0435), and TP47 (Tp0574) recombinant antigens to detect IgG antibodies, and used the T. pallidum Particle Agglutination assay (TPPA) as a reference for accuracy statistics analysis. Corroborating with the results of our study, TP15 obtained high accuracy performance, with a sensitivity of 91.2% and specificity of 99.0%, and Tp0435 presented the optimal accuracy performance, with a sensitivity of 96.9% and specificity of 99.0%, while those of Tp0574 were 98.8% and 98.0%, respectively.^18^ However, our results showed that CE*Tp*0574 has inferior sensibility than LISA-TP47 (Tp0574),^18^ which might be due to the difference between LISA and ELISAs, since LISA was reported as 10^4^-fold more sensitive than ELISA when detecting anti-HIV-1 p24 antibodies.^51^ Nonetheless, previous works using ELISA demonstrated that the protein exhibits a sensitivity range of 82.1% to 100% and a specificity of 100%,^46,52^ supporting our results and its potential use in syphilis screening in clinical laboratories. Tp0574 protein was also recently reported as highly immunogenic and critical for syphilis serodiagnosis and has been proven as a key antigen recognized by the immune system during infection.^46,52^ Tp0574 is the most abundantly expressed T. pallidum-specific protein, playing a functional role in inflammatory responses and syphilis pathology.^52^ These findings enhance the understanding of syphilis-related inflammation and corroborate our results, which also confirm the high immunogenicity of Tp0574 by anti-CE*Tp*0574 polyvalent detection. Other recent studies^15,25,46^ support our findings, highlighting the importance of Tp0435 in serodiagnosis in treponemal tests for syphilis.

The association of Tp0171 and Tp0435 proteins in ELISA achieved more favorable diagnostic accuracy parameters when compared with other serodiagnosis methods in patients with syphilis. Random anti-IgG treponemal EIA screening of 1,822 NDS identified four positive samples missed by VDRL, which were also undetected by two T. pallidum hemagglutination (TPHA) tests, highlighting the detection of specific pathogenic antibodies. Western blot (immunoblot) technology confirmed EIA-positive samples, showing reactivity with at least three T. pallidum bands (47, 30, and 15.5 kDa).^36^ Syphilis Fast, a latex test, which uses a pool of recombinant T. pallidum antigens proteins (Tp0171, Tp0435, and Tp0574), reported significantly higher specificity (99.8%) and sensibility (93.8%) when compared to VDRL (99.1% specificity and 46.5% sensibility).^37^ An ELISA based on the Tp0171, Tp0435, and Tp0574 (TpN15–17–47) fusion protein achieved a positivity rate of 99.5% in syphilis patient samples, outperforming traditional methods such as TPHA and TRUST.^53^

The progress in T. pallidum genome sequencing has improved the production of recombinant antigens for diagnostics, particularly immunodominant lipoproteins, such as Tp0171, Tp0435, and Tp0574. Many FDA-approved treponemal tests using these antigens achieve 95%–99% sensitivity and specificity. However, challenges remain in detecting the disease during the early and late stages, when humoral immunity to T. pallidum is insufficiently developed for reliable detection. Sensitivity could be improved by incorporating new treponemal antigens that complement immunodominant targets, supporting the need to explore novel proteins and further investigate previously studied antigens.^25^

The findings of our study were based on an assay that can concomitantly detect polyvalent antibodies (IgA, IgM, and IgG) in serum samples from individuals with different clinical stages of syphilis. The two peptides (CE*Tp*0171 and CE*Tp*0435) were able to identify and distinguish between positive and negative sera in distinct phases of the disease, showing the potential application of these antigens for different immunoglobulin detection, which may support the indication of different stages of the infection, principally on early stages.^54^ These peptides, especially CE*Tp*0435, also demonstrated high accuracy as a serological panel for multistage syphilis, successfully detecting the primary, secondary, and latent stages with an AUC of 1.0, and 100% sensitivity and specificity (CE*Tp*0435, Figure 5). CE*Tp*0171 demonstrates strong performance in detecting secondary syphilis (AUC = 1.0, 100% sensitivity and specificity, Figure 4C) and good accuracy for primary (AUC = 0.9856, 87.50% sensitivity, 97.44% specificity, Figure 4B) and latent stages (AUC = 0.9808, 83.33% sensitivity, 97.44% specificity, Figure 4D). However, CE*Tp*0574 showed limitations in accurately detecting positive rates across all syphilis stages (Figure 6A) but demonstrated sufficient performance for the primary (AUC = 0.9754, 85.71% sensitivity, 100% specificity, Figure 6B), secondary (AUC = 0.8756, 83.33% sensitivity, 96.55% specificity, Figure 6C), and latent stages (AUC = 0.8726, 65.22% sensitivity, 96.55% specificity, Figure 6D).

According to previous work on Tp0171, Tp0435, and Tp0574 proteins, each of them has specific characteristics, and antibody profiles against these proteins, IgM and IgG, present different expression patterns in different syphilis clinic stages. IgM antibodies against Tp0171 are mainly detected in the latent phase.^39^ Antibodies against Tp0574 are detected in all syphilis stages,^12,15,53^ underscoring its importance in disease pathogenesis.

Both peptides CE*Tp*0171 and CE*Tp*0435 were designed using a bioinformatics tools pipeline,^32^ based on Tp0171 and Tp0435 epitope regions (Figure 2 and Table 2). These, as recombinant proteins, were also used before as satisfactory antigens to evaluate syphilis treatment efficacy since antibodies anti-TP0171 and TP0435 declined in sera samples.^18,25^ It should be noted that these studies also evaluated the same treatment approach that we used in our study. Thus, the treatment with benzathine penicillin inhibited the serum antibody response to TP0171 (TP15) and TP0435 (TP17), which could be detected by CE*Tp*0171 (Figure 9A) and CE*Tp*0435 (Figure 9B). Haynes et al.^25^ demonstrated significant reactivity of the Tp0435 antigen across different syphilis stages and observed a reduction in reactivity post-treatment, suggesting its potential as a biomarker for monitoring treatment response. A significant decline in IgG levels was observed following antisyphilitic treatment, emphasizing the utility of Tp0171 for monitoring treatment response.^39^ However, seven individuals from the post-treatment group remain seropositive in the chemiluminescence treponemal test (TT) despite receiving adequate treatment and having no active infection. Treponemal tests, currently available, typically remain positive for life, even after successful treatment,^55,56^ indicating past infection rather than ongoing disease. In this context, our peptide-based platform demonstrated effectiveness as a treponemal test for monitoring treatment, as none of the seven participants with a history of syphilis showed reactivity. Furthermore, no reactivity was observed in individuals undergoing treatment or post-treatment (Figure 9), reinforcing the potential of this approach for differentiating past infections from active disease.

Considering the significant impact of misdiagnoses on patient outcomes and the critical dependence of therapeutic interventions on diagnostic accuracy, the prototype peptide-based diagnostic platform was evaluated through a cross-sectional study of diagnostic accuracy. The accuracy study allowed us to assess the ability of the index test to correctly classify study participants as having syphilis or not and to correctly predict the event or condition, i.e., presence or absence of infection, in the future.^57^ One of the main challenges in developing serologic tools is achieving a high analytical performance with complex biological samples, such as patient serum, which contains numerous proteins that may interfere with the assay. Our platform reached a Technology Readiness Level (TRL) of 5–6, as prototype diagnostic platform peptide-based enzyme-linked immunosorbent assays using five peptides derived from highly immunogenic T. pallidum proteins epitopes were tested using a biobank of 122 human sera samples simulating a relevant environment.^58^

In addition, according to the WHO, the major barrier to STIs control and prevention is the absence of reliable, low-cost point-of-care tests (POCTs) that facilitate both diagnosis and treatment within a single patient visit.^59^ This issue is particularly critical for syphilis, where early and accurate detection is essential to reducing transmission and improving clinical outcomes. The Global Health Sector Strategy for the Control and Prevention of STIs highlights the potential of POCTs to revolutionize STI management by improving diagnosis, treatment, and surveillance across the care continuum. This aligns with the current Technology Readiness Level (TRL) of 5–6 for the peptide-based ELISA, underscoring its potential to bridge gaps in STI diagnostics. Continued development of this platform could position it as a key innovation in global syphilis control efforts, advancing healthcare accessibility and equity, in alignment with Sustainable Development Goal 3 (SDG 3), which aims to ensure healthy lives and promote well-being for all at all ages.^60^

## 4 Limitations

This study presents some limitations. All ELISAs of CE*Tp*0171 and CE*Tp*0435 were performed with *o*-phenylenediamine (OPD) as the detection reagent, while CE*Tp*0574, CE*Tp*0684, and CE*Tp*0453 were carried out with tetramethylbenzidine (TMB) as the detection reagent. This change in the ELISA detection reagent may have been introduced, potentially impacting the peptide performance results, since mainly protein Tp0574 is reported as a key antigen for syphilis serodiagnosis.^52,53^ TMB has been reported to exhibit greater sensitivity than that of the OPD, producing the highest signal. However, despite its widespread application in peroxidase-like enzyme detection, TMB presents a critical limitation: its lack of specificity, which can complicate the control of nonspecific background signals in negative samples.^61^ Otherwise, both TMB and OPD substrates demonstrated superior analytical performance in terms of the limit of detection (LOD) and limit of quantification (LOQ), dynamic range, and sensitivity.^62^ All samples were previously diagnosed using the reverse algorithm; please see Figure 1. The treponemal chemiluminescence test, used for syphilis screening at the Rio Verde/GO—Brazil, was based on TP recombinant antigens—Tp0171 (Tp15), Tp0435 (Tp17), and Tp0574 (Tp47)—and used the full-length forms of these three major antigens as a comparative reference. However, some of our limited results for peptides CE*Tp*0684 and CE*Tp*0453 could be related to our reference test, in which none of those have been based on full-length Tp0684 and Tp0453 proteins. Additionally, the small sample sizes for the primary (*n* = 7) and secondary (*n* = 6) stages and the absence of patients in the late latent stage limit the generalizability of the findings across all stages of syphilis. The limited sample size of HIV-coinfected and syphilis history participants may have influenced the findings, as it precluded specific analyses to determine the potential impact of coinfection and syphilis history on syphilis diagnostic outcomes. These factors should be addressed in future studies to enhance the robustness and applicability of the diagnostic prototype platform. From a further perspective, these peptides could be evaluated by comparing them with reference full-length proteins. Also, future studies are needed to investigate the HIV-coinfected and syphilis history on our diagnostic prototype platform.

## 5 Conclusion

We developed a treponemal test prototype diagnostic platform peptide-based for syphilis diagnosis that was validated by a cross-sectional study. The CE*Tp*0435 peptide demonstrated outstanding diagnostic performance, achieving 100% sensitivity and specificity, AUC = 1.0. Peptides CE*Tp*0171 and CE*Tp*0574 also exhibited a high diagnostic accuracy. Although CE*Tp*0684 and CE*Tp*0453 showed moderate performance, CE*Tp*0453 presented limited specificity. The epitopes mimetic peptides applied on peptide-based ELISA provide the benefit of semiquantitative detection of syphilis antibodies using human samples, which can be valuable for tracking antibody titers against treponemal immunodominant antigens, as well as for assessing the effectiveness of the syphilis therapy approach.

For further perspectives, we suggest that these peptides be evaluated on other syphilis patient biobanks to enhance their robustness across all stages of syphilis. Further, these peptides can be adjusted into rapid immunoassay diagnostics, following WHO-recommended REASSURED (Real-time connectivity, Ease of specimen collection, Affordable, Sensitive, Specific, User-friendly, Rapid and robust, Equipment-free or simple, and Deliverable to end-users) criteria for point-of-care tests. The identification of late latent infections at early stages as well as the epidemiology can play an important role in the treponemal test for syphilis diagnosis.

## 6 Materials and Methods

### 6.1 Study Design

A diagnostic accuracy cross-sectional study was performed in order to validate a prototype technology as a serodiagnosis platform for syphilis, herein defined as the index test.^57^ The set of tests, defined as the diagnostic algorithm for syphilis, is the gold standard diagnostic algorithm, which was used as a reference standard to estimate the accuracy, sensitivity, and specificity of the index test. The study was completed following the provisions of the Standards for Reporting Diagnostic Accuracy Studies guidelines.^63^

### 6.2 Selection of Participants

Participants were sampled through a cross-sectional study and organized into participant flowcharts by using a diagram. Baseline demographic and clinical characteristics of participants were determined, as well as the distribution of clinical course and severity in those participants with the target condition, i.e., positive syphilis determined by the positive reference test result/event (cases).^64^ Thus, the participants’ epidemiological profile was determined to assess the accuracy, sensitivity, specificity, and predictive values of the index test as a surveillance tool for exposure to syphilis, supporting the study of diagnostic accuracy.^32^

#### 6.2.1 Ethical Approvals

This study was approved by the Research Ethics Committee of Universidade de Rio Verde (UniRV) under protocol number CAAE: 59241322.0.0000.5077. Also, this study was carried out in accordance with the Declaration of Helsinki, with respect to the ethical principles for research involving humans. The samples were collected with written consent from all patients, who signed the *Written Free and Informed Consent Form* previously approved by the Research Ethics Committee of UniRV.

#### 6.2.2 Inclusion and Exclusion Criteria

The inclusion criteria were based on individuals between 18 and 80 years old, of any sex, who had undergone a diagnostic reference test for syphilis, following the screening and diagnostic confirmation algorithm associated with the disease clinical stage and who agreed to participate as volunteers in this study, signing the *Written Free and Informed Consent Form*.

Individuals with incomplete clinical or laboratory information that precludes accurate diagnostic classification or those with health conditions that impair their understanding or proper participation in the study were excluded from the investigation.

##### 6.2.2.1 Sample Collection and Syphilis Testing

Participant samples were collected from June 2022 to July 2023. Peripheral human blood samples were collected from each volunteer by venipuncture. The samples were centrifuged at room temperature for 10 min at 2,000 g to obtain the serum. Following centrifugation, the sera were immediately transferred to a clean microcentrifuge. The sera samples were stored at −80 °C for further analysis.

The reverse approach is used for the diagnostic confirmation algorithm at Rio Verde *Testing and Counseling Center* (from Portuguese: Centro de Testagem e Aconselhamento—CTA)—Goiás, Brazil, following the Technical Manual for the Diagnosis of Syphilis from the Brazilian Ministry of Health.^21^ Thus, the procedure to confirm syphilis was the treponemal serological test, chemiluminescence, followed by a nontreponemal test. The screening treponemal-specific test used during the study period was chemiluminescence; the venereal disease research laboratory (VDRL) was used as a nontreponemal test. When the treponemal test is reactive but the VDRL is not, a rapid plasma reagin test (RPR) is used, as another treponemal test. In Rio Verde CTA, VDRL is also applied as a prognostic monitoring test to evaluate the patient treatment. Patients were treated with benzylpenicillin benzathine 2.4 million I.U. intramuscularly in a single dose. The flowchart of the diagnostic confirmation algorithm at the Rio Verde CTA is illustrated in Figure 1.

### 6.3 Design of the Syphilis Serodiagnosis Platform Index Test

#### 6.3.1 T. pallidum Antigenic Proteins Epitope Prediction

##### 6.3.1.1 Proteins Selection Criteria

The Tp0171 (Tp15), Tp0435 (Tp17), Tp0574 (Tp47), Tp0684, and Tp0453 proteins were selected based on the following criteria: immunogenic and antigenic extensively validated in immune proteomic studies, applied in current treponemal tests for syphilis screening,^12,15,22–25^ and are also available in biological databases, such as GenBank and/or PDB.

##### 6.3.1.2 Sequence Alignment

The primary sequences of protein Tp0171 (PDB: 4XDU), Tp0435 (PDB: 4U3Q), Tp0574 (PDB: 1O75), Tp0453 (PDB: 3K8J), and Tp0684 (PDB: 5JX2) from T. pallidum were analyzed using the BLAST-p algorithm against proteins from other Treponema spp. that could present cross-reactions, such as the etiological agents of endemic treponematoses: bejel, pinta, and yaws, which were deposited in databases such as GenBank. Then, these proteins were aligned by the Clustal Omega server^65^ with other primary protein sequences that potentially induce a nonspecific immune response in patients with syphilis.^9,10^

##### 6.3.1.3 Specific B-Cell Epitope Prediction

The epitopes were predicted using the Epitope Prediction algorithm of the Immune Epitope Database (IEDB). This server has a series of algorithms aimed at predicting B-cell epitopes. The Emini Surface Accessibility algorithm, used to identify amino acids in the primary sequence that are most exposed on the protein surface, and the BepiPred Linear Epitope Prediction algorithm, which predicts surface residues most likely to be recognized by antibodies, were both applied. The spatially close amino acids in the tertiary structures were used to design a peptide that mimicked the epitopic region, as previously described.^66^ To obtain the three-dimensional structures, protein research was performed by selecting the Protein Data Bank (PDB) database from which the three-dimensional structure with the best resolution was selected. Epitopes were designed by combining amino acids from different protein regions and mapping them onto the 3D structures collected in the PDB or created via structural modeling.^67^ The design of epitopes was performed using the SwissPDB-viewer.^32,68^

#### 6.3.2 FMOC Solid-Phase Peptide Synthesis and Characterization

The peptide sequences of epitopes were manually synthesized using the FMOC Solid-Phase Peptide Synthesis technique, as previously described.^32^ Then, these peptides were characterized by electrospray mass spectrometry using a Xevo TQ-S micro-Triple Quadrupole spectrometer coupled to a UPLC system (Waters Corporation, Framingham, MA, USA). The peptide samples were diluted in acetonitrile with 0.1% formic acid and infused in positive ionization mode with argon collision fragmentation. The mass spectra were analyzed by MassLynx software, version v4.2 SCN1001 (2019, Waters Inc.). Physicochemical parameters of the peptides were analyzed using PepCalc and ExPASy—Compute pI/Mw,^30,32^ and the peptide identity was confirmed by the mass spectra fragmentation profile.

#### 6.3.3 Peptide-Based ELISA

Designed synthetic epitopes were used as antigens for syphilis-specific antibodies screening by peptide-based ELISA, performed according to refs 30 and 32 with some adaptations. High-binding EIA 96-well plates (Costar 3590, Corning) were coated with 100 μL/well of peptides (individually, 0.05 μg·μL^–1^) in carbonate buffer (pH 9.6) and incubated overnight at 4 °C. The plates were washed with phosphate buffer saline (PBS) containing 0.05% Tween 20 (PBS-T) and blocked with a 5% bovine serum albumin (BSA) blocking solution at 37 °C for 1 h. After washing, serum samples diluted (1:100) in a solution of 0.5% BSA in PBS-T were added to each well (100 μL/well) and incubated at 37 °C for 1 h. The wells were washed and incubated with polyvalent antihuman IgA, IgM, and IgG conjugated with peroxidase (Sigma-Aldrich) at 37 °C for 1 h, at an optimal dilution (1:5000), according to previous standardization tests. After another wash, colorimetric detection was performed using *o*-phenylenediamine (OPD) (0.2 μg/μL in citrate buffer, pH 5.2) with 3 μL of hydrogen peroxide or with 3,3′,5,5′-tetramethylbenzidine (TMB), and the reaction was stopped with 2 M sulfuric acid. Optical density was measured at 492 nm (OPD) or 450 nm (TMB) using a SpectraMax M3 spectrophotometer. Preliminary titration curves were performed to optimize antigen, sample, and conjugate concentrations. In order to reduce analytical variation, the experiments were performed using sera samples with the same reagents (lots, dilutions, etc.). Analytical robustness was guaranteed by performing two different peptide-based ELISA sessions interday, using the same sera samples tested in the two independent experiments for all peptides individually. Bland–Altman plot analysis (data not shown) indicated that differences in optical density (OD) values between the duplicates did not increase with the variation of their serological mean. Thus, the data analyses were performed by serological means from each serum sample against each peptide as coating ELISA antigen.

#### 6.3.4 Data Analyses

For the accuracy study, data analyses were conducted, the cross-tabulation method (contingency table), and accuracy statistics were estimated using the receiver operating characteristics (ROC) curve analysis method. The cutoff values to differentiate negative and positive samples against each antigen were determined considering the mean of negative control samples (uninfected) plus 2.5 SD (standard deviation). Sensitivity (Se), specificity (Sp), accuracy—area under the curve (AUC), positive predictive value (PPV), negative predictive value (NPV), and likelihood ratio (LR) of the antigens were determined from the ROC curves with confidence intervals (CI) using a 95% confidence level (95% CI), for each antigen.^29,30,32^

As a means to validate the peptide-based platform as a tool for syphilis treatment efficacy, normality tests were applied, followed by tests for equality of variances and tests to evaluate the significance of differences between groups and posthoc for multiple comparisons.^69^ The normality of the data was tested using the Shapiro–Wilk test. In one of the groups, the Shapiro–Wilk test indicated *p* < 0.001, suggesting a significant violation of normality. Deviations were also observed in the Q–Q plot (Figure S1), confirming the non-normality. The Brown–Forsythe test was performed for the homogeneity of variances between the groups. It is more robust to non-normal distributions and uses the median, making it more suitable for data with skewness. Analysis of variance was performed using Welch’s ANOVA since this test was based on the violation of normality within a group and the heterogeneity of variances between groups. For multiple comparisons between experimental groups, Dunnett’s T3 test was used, which is appropriate for unequal variances and different sample sizes. *P*-values ≤0.05 were considered statistically significant. Statistical analyses were performed using GraphPad Prism version 10.4.1 software.

## Supplementary Material



## Notes

Statistical analyses to validate the peptide-based platform as a tool for syphilis treatment efficacy; normality tests applied using the Shapiro–Wilk test, which indicated *p* < 0.001, suggesting a significant violation of normality; normal quantile–quantile (QQ) plot to confirm the non-normality, the results of which justify the application of robust statistical methods for non-normal data; and monoisotopic mass and fragmentation pattern of each peptide to foment the mass spectrometry characterization of the synthetic peptides (PDF)

## Abbreviations

AUC: area under the curve
BSA: bovine serum albumin
CI: confidence intervals
CTA: Testing and Counseling Center (from Portuguese: Centro de Testagem e Aconselhamento)
EIA: immunosorbent assay
ELISA: enzyme linked immunosorbent assay
HIV: human immunodeficiency virus
IEDB: immune epitope database
LISA: linked immunosorbent assay
LOD: limit of detection
LOQ: limit of quantification
NPV: negative predictive value
NTTs: nontreponemal tests
OD: optical density
OMP: outer-membrane proteins
OPD: *o*-phenylenediamine
PBS: phosphate buffer saline
PBS-T: phosphate buffer saline with Tween 20
PDB: Protein Data Bank
PPV: positive predictive value
ROC: receiver operating characteristics
RPR: rapid plasma reagin
SD: standard deviation
SDG 3: Sustainable Development Goal 3
Se: sensitivity
Sp: specificity
STI: sexually transmitted infection
TMB: 3,3 ´,5,5 ´-tetramethylbenzidine
TPHA: Treponema pallidum hemagglutination
TPPA: Treponema pallidum particle agglutination
TRL: technology readiness level
TTs: treponemal tests
UPLC: ultra-performance liquid chromatography
VDRL: venereal disease research laboratory
WHO: World Health Organization
