# Supplementary material for: Design of Epitopes from Treponema pallidum Lipoprotein Antigens for Syphilis Diagnosis and Treatment Prognosis
Source: ACS Infect Dis. 2025 May 23;11(6):1606–22. doi: 10.1021/acsinfecdis.5c00155 (PMC12172048; doi:10.1021/acsinfecdis.5c00155)
Supplement: Supplementary file 1 [file id5c00155_si_001.pdf]

Supporting Information

Design of epitopes from *Treponema pallidum* lipoprotein-antigens for syphilis diagnosis and treatment prognosis

Letícia Alves Borghezan[†]; Lara Cândida de Sousa Machado[†,‡]; Iara Barreto Neves Oliveira [γ]; Mírian Ívens Fagundes[†]; Nicoly Silveira Apolidório[†]; Renato Canevari Dutra da Silva[‡]; Victor Garcia Freire[†,‡]; Antônio Augusto Schäfer[‡,§]; Rahisa Scussel[†,‡+]; Ricardo Andrez Machado-de-Ávila[†,‡+*]

[†]Programa de Pós-Graduação em Ciências da Saúde, Universidade do Extremo Sul Catarinense. Universitário, Postal code: 88806-000. City: Criciúma. State: Santa Catarina, Brazil.

[‡]Faculdade de Medicina, Universidade de Rio Verde. Fazenda Fontes do Saber, Campus Universitário, 75901-970, Rio Verde, Goiás, Brazil.

[γ]Faculdade de Medicina, Universidade de Rio Verde. Avenida T-13 Qd. S-06, Lts.08/13. Setor Bela Vista, 74823-440, Goiânia, Goiás, Brazil.

[§]Programa de Pós-Graduação em Saúde Coletiva, Universidade do Extremo Sul Catarinense. Universitário, 88806-000, Criciúma, Santa Catarina, Brazil.

[+]*These authors contributed equally as senior authors.*

[*]Corresponding author

Dr. Ricardo Andrez Machado-de-Ávila. **Address:** Laboratório de Fisiopatologia Experimental do Programa de Pós-Graduação em Ciências da Saúde, Universidade do Extremo Sul Catarinense, Universitária Avenue, 1105, S Building, Room 017, 88806-000, Criciúma, Santa Catarina, Brazil.

Phone number: +55 48 3431-2773

E-mail address: r_andrez@yahoo.com.br

Figure S1 – Normal QQ (Quantile-Quantile) plot of data from surveilling syphilis treatment efficacy.

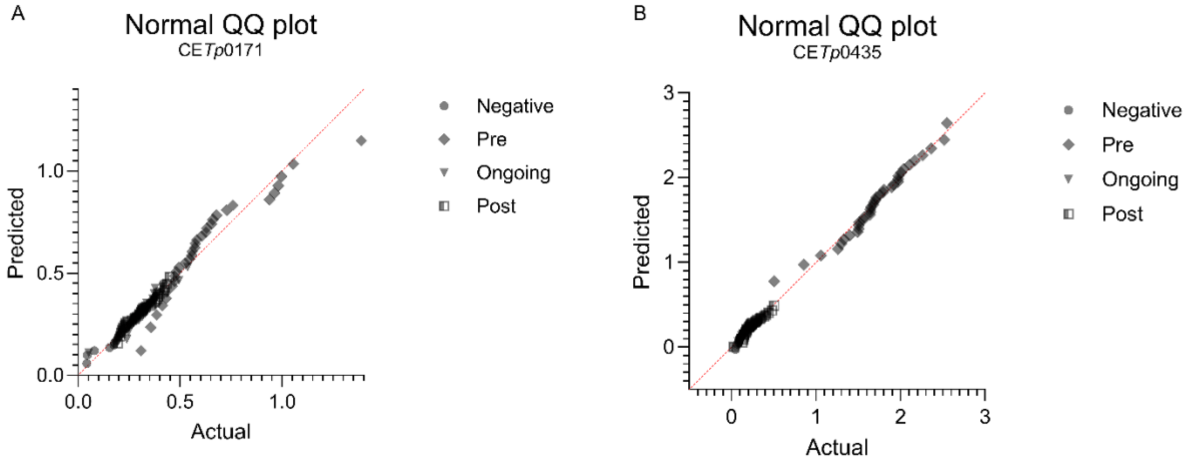

A) Distribution representation of CE*Tp*0171 data about the theoretical normal distribution. While B) represents the distribution of CETp0435 data about the theoretical normal distribution. It can be observed that for most groups of both peptides A and B, the points follow the reference line, indicating adherence to normality. However, group pre-treatment presented deviations, confirmed by the Shapiro-Wilk normality test ($p < 0.001$). These results justify the application of robust statistical methods for non-normal data, such as Brown-Forsythe followed by Welch's ANOVA.

Table S1 – Molecular weight determination from monoisotopic mass and fragmentation pattern for the synthetic peptides

Peptide	Molecular formula	Isotropic mass(g/mol)	Fragment ions (m/z)
CE*Tp*0171	$C_{131}H_{199}N_{41}O_{38}$	2956.27	985.84 (+3)
			739.63 (+4)
			591.90 (+5)
			493.42 (+6)
			423.08 (+7)
			370.32 (+8)
CE*Tp*0435	$C_{80}H_{134}N_{24}O_{26}$	1848.09	1847.99 (1+)
			924.50 (2+)
			616.67 (3+)
			462.75 (4+)
			370.40 (5+)
			308.83 (6+)
			264.86 (7+)
CE*Tp*0574	$C_{123}H_{196}N_{36}O_{43}$	2867.12	1433.72 (+2)
			956.15 (+3)

			717.36 (+4)
			574.09 (+5)
			478.57 (+6)
CE*Tp*0684	$C_{77}H_{117}N_{23}O_{28}$	1812.91	906.92 (+2)
			604.95 (+3)
			453.96 (+4)
			362.37 (+5)
			302.98 (+6)
			259.84 (+7)
CE*Tp*0453	$C_{102}H_{162}N_{30}O_{35}S_1$	2400.65	2400.16 (1+)
			1200.58 (2+)
			800.72 (3+)
			600.79 (4+)
			480.84 (5+)
			400.87 (6+)
			343.74 (7+)
